# Sex-Dependent Mediation of Leptin in the Association of Perilipin Polymorphisms with BMI and Plasma Lipid Levels in Children

**DOI:** 10.3390/nu14153072

**Published:** 2022-07-26

**Authors:** Claudia Vales-Villamarín, Jairo Lumpuy-Castillo, Teresa Gavela-Pérez, Olaya de Dios, Iris Pérez-Nadador, Leandro Soriano-Guillén, Carmen Garcés

**Affiliations:** 1 Lipid Research Laboratory, IIS-Fundación Jiménez Díaz, 28040 Madrid, Spain; claudia.vales@quironsalud.es (C.V.-V.); olaya.dios@quironsalud.es (O.d.D.); iris.perezn@quironsalud.es (I.P.-N.); 2 Laboratory of Diabetes and Vascular Pathology, IIS-Fundación Jiménez Díaz, UAM, 28040 Madrid, Spain; jairo.lumpuy@estudiante.uam.es; 3 Department of Pediatrics, IIS-FJD, 28040 Madrid, Spain; TGavela@fjd.es (T.G.-P.); LSoriano@fjd.es (L.S.-G.)

**Keywords:** PLIN polymorphisms, leptin, BMI, NEFA, HDL-cholesterol, Apo A-I

## Abstract

Variations in the perilipin (*PLIN*) gene have been suggested to be associated with obesity and its related alterations, but a different nutritional status seems to contribute to differences in these associations. In our study, we examined the association of several polymorphisms at the *PLIN* locus with obesity and lipid profile in children, and then analyzed the mediation of plasma leptin levels on these associations. The single-nucleotide polymorphisms (SNPs) rs894160, rs1052700, and rs2304795 in *PLIN1*, and rs35568725 in *PLIN2*, were analyzed by RT-PCR in 1264 children aged 6–8 years. Our results showed a contrasting association of *PLIN1* rs1052700 with apolipoprotein (Apo) A-I levels in boys and girls, with genotype TT carriers showing significantly higher Apo A-I levels in boys and significantly lower Apo A-I levels in girls. Significant associations of the SNP *PLIN2* rs35568725 with high-density lipoprotein cholesterol (HDL-cholesterol), Apo A-I, and non-esterified fatty acids (NEFA) were observed in boys but not in girls. The associations of the SNPs studied with body mass index (BMI), NEFA, and Apo A-I in boys and girls were different depending on leptin concentration. In conclusion, we describe the mediation of plasma leptin levels in the association of SNPs in *PLIN1* and *PLIN2* with BMI, Apo A-I, and NEFA. Different leptin levels by sex may contribute to explain the sex-dependent association of the *PLIN* SNPs with these variables.

## 1. Introduction

Perilipins are proteins that coat intracellular lipid droplets [1,2] and play a central role in lipid storage and mobilization. Non-phosphorylated perilipin protects the lipid core from the activity of lipases, such as hormone-sensitive lipase (HSL), which hydrolyze triglycerides into glycerol and fatty acids, preventing basal lipolysis and promoting cellular triglyceride storage by limiting lipase access to triglyceride stores [3,4,5]. Once phosphorylated, however, perilipin allows lipases to access lipid droplets and, hence, causes active lipolysis. Thus, the activity of perilipin may play a role in body weight and lipid metabolism by regulating adipocyte metabolism, fat storage, and lipolysis [6].

The most widely studied member of the family, perilipin 1 (PLIN1), is the most abundant protein on the surface of adipocyte lipid droplets and the major substrate for the cAMP-dependent protein kinase [7]. The human *PLIN1* gene is found at chromosomal location 15q26.1 [8]. It has been shown to be a susceptibility locus for obesity and hypertriglyceridemia [9]. In fact, some studies have shown that common polymorphisms in the *PLIN1* gene are associated with obesity risk and obesity-related phenotypes [10,11,12]. Furthermore, *PLIN1* single-nucleotide polymorphisms (SNPs) have also been related to variability in weight loss [12,13,14,15]. However, several analyses of the associations between these polymorphisms and body weight and BMI have reported divergent results [16]. A polymorphism in *PLIN2*, another member of the family involved in the formation of lipid droplets, has also been shown to affect the structure and function of the protein. A substitution of serine by proline at the 251 position results in an altered protein structure and a reduction in lipolysis and plasma triglycerides [17,18]. 

To our knowledge, no studies have investigated the association of the *PLIN* SNPs in a general population of children. A couple of studies in children have focused on specific populations, such as obese children or children in a weight loss intervention [19,20]. Thus, limited evidence is available for Caucasian pre-pubertal children regarding the possible association of these SNPs with obesity-related alterations.

Significant gene–diet interactions involving these *PLIN* SNPs have been reported [21,22,23,24], suggesting that nutritional status may modify the association of *PLIN* polymorphisms with these traits. Leptin levels can be considered a good indicator of nutritional status [25]. 

Leptin, a hormone consistently related to obesity and obesity-related alterations [26], has been shown to exert direct and indirect effects on adipocyte metabolism [27]. As adipocytes express leptin receptors, leptin may influence adipocyte metabolism directly, triggering lipolysis via a lipolytic pathway mediated by cAMP, protein kinase A, perilipin, and HSL. Indeed, cAMP activates the protein kinase A, which is then able to phosphorylate cellular proteins, such as perilipin and HSL. Phosphorylated perilipin may activate HSL function, hydrolyzing triglycerides into glycerol and fatty acids [28,29].

In our study, we aimed to investigate, the association of body mass index (BMI) and plasma lipid levels with several *PLIN1* SNPs (11482G > A (rs894160), 13041A > G (rs2304795), and 14995A > T (rs1052700)) and with the *PLIN2* SNP Ser251Pro (rs35568725) in a large, population-based cohort of Spanish prepubertal children aged between 6 and 8 years. The SNPs selected have been associated with obesity-related phenotypes in adults, but they have not been previously studied in a cohort of children. In addition, we aimed to explore whether leptin modulates the effect of these polymorphisms.

## 2. Materials and Methods

### 2.1. Subjects

Our study comprised a population of 1264 children, aged 6–8 years (633 boys and 631 girls), who participated of the Four Provinces Study (4P Study), a cross-sectional study aiming to examine cardiovascular risk factors in Spanish children [30]. All children with a parent-reported chronic disease were excluded. Parents or legal guardians were required to provide written informed consent for their children to participate in the study. The study protocol complied with the Helsinki Declaration guidelines and was approved by the Clinical Research Ethics Committee of the IIS-Fundación Jiménez Díaz (PIC016-2019 FJD).

### 2.2. Anthropometric Data

Measurements were taken with the children lightly dressed and barefoot as previously described [30]. Height was measured to the nearest millimeter using a portable stadiometer, weight was recorded to the nearest 0.1 kg using a standardized electronic digital scale, and body mass index (BMI, weight in kilograms divided by height in meters squared, kg/m^2^) was calculated from these parameters.

### 2.3. Biochemical Data

Fasting (12 h) blood samples were obtained by venipuncture and centrifuge. Serum and plasma samples were separated and stored at −70 °C. Biochemical determinations were performed as previously described [30]. Cholesterol and triglyceride (TG) concentrations were measured enzymatically (Menarini Diagnostics, Florence, Italy) in a RA-1000 Autoanalyzer (Technicon Ltd., Dublin, Ireland). High-density lipoprotein cholesterol (HDL-cholesterol) was measured after precipitation of apolipoprotein B-containing lipoproteins with phosphotungstic acid and Mg (Roche Diagnostics, Madrid, Spain). Plasma apolipoprotein A-I (Apo A-I) and apolipoprotein B (Apo B) concentrations were measured by immunonephelometry (Dade Berhing, Frankfurt, Germany). Low-density lipoprotein cholesterol (LDL-cholesterol) was calculated according to the Friedewald formula. Non-esterified fatty acids (NEFA)were measured by using the Wako NEFA-C kit (Wako Industries, Osaka, Japan). Leptin concentrations were determined by ELISA using a commercial kit (Leptin EIA-2395, DRG, Marburg, Germany), as described elsewhere [30].

### 2.4. Genotyping Assays of SNPs in PLIN1 and PLIN2

All DNA was isolated from 10-mL EDTA-blood samples according to standard procedures. To determine the polymorphism in the perilipin genes, the following predesigned TaqMan SNP Genotyping Assays from Applied Biosystems (Waltham, MA, USA) were used: C_8722593_10, C_8722587_10, and C_9304320_20 for the SNPs in *PLIN1* rs894160, rs1052700, and rs2304795, respectively, and C_25764255_10 for the SNPrs35568725 in *PLIN2*. A StepOnePlus™ Real-Time PCR System (Applied Biosystems) was used for allelic discrimination. Additionally, PCR was performed with a mixture of 10 ng of genomic DNA, TaqMan^®^ SNP Genotyping Assay (20X), and TaqPath™ ProAmp™ Master Mix (Applied Biosystems). Samples were cycled under the following recommended conditions: 95 °C for 10 min, 95 °C for 15 sec, and 60 °C for 1 min, repeated over 40 cycles.

### 2.5. Statistical Analysis

Statistical analyses were performed using the SPSS software package, version 26.0 (IBM, New York, NY, USA) and the GraphPad Prism statistical software (San Diego, CA, USA, Version 8). The normality of quantitative variables was analyzed by the Kolmogorov–Smirnov test, revealing a non-parametric distribution. The Mann–Whitney U test was used to perform sex-based comparisons of median BMI values, lipid profile variables (TC, TG, LDL-cholesterol, Apo B, HDL-cholesterol, Apo A-I, NEFA), and leptin. Differences in median values for the variables under study according to the different genotypes of the SNPs studied were tested using the Mann–Whitney or Kruskal–Wallis tests in our population, divided by sex and grouped according to median plasma leptin levels in each sex (2.26 ng/mL in boys and 5.25 ng/mL in girls).

## 3. Results

Table 1 shows the anthropometric and biochemical data of the children according to sex. The mean age was similar in boys and girls. Plasma concentrations of HDL-cholesterol and Apo A-I were significantly higher, and concentrations of TG, LDL-cholesterol, and Apo B were significantly lower, in boys compared to girls. Leptin levels were significantly (*p* < 0.001) higher in girls.

The frequencies of the genotypes and alleles for the SNPs studied are shown in Table 2. The genotype distributions were within the Hardy–Weinberg equilibrium. These frequencies were similar to those described in other Caucasian populations. 

When analyzing the relationship of the *PLIN1* SNPs rs894160, rs2304795, and rs1052700 with the variables under study by sex (Table 3), a significant and opposite association was discovered between *PLIN1* rs1052700 and Apo A-I levels between boys and girls. The TT carriers showed significantly higher Apo A-I levels as compared with CC and CT carriers in boys, while significantly lower Apo A-I levels were observed in girls. When analyzing the SNP of *PLIN2* rs35568725 (Table 3), carriers of the AG and GG genotypes were grouped together due to the small number of children who were homozygous for the less common allele (G). Significant differences between AA carriers and carriers of the G allele were observed for HDL-cholesterol (*p* = 0.005), Apo A-I (*p* = 0.009), and NEFA (*p* = 0.002) concentrations in boys, though no such differences were observed in girls. No significant associations were detected between the PLIN polymorphisms and LDL-cholesterol, Apo B, or leptin levels (data not shown).

**Figure 1:**
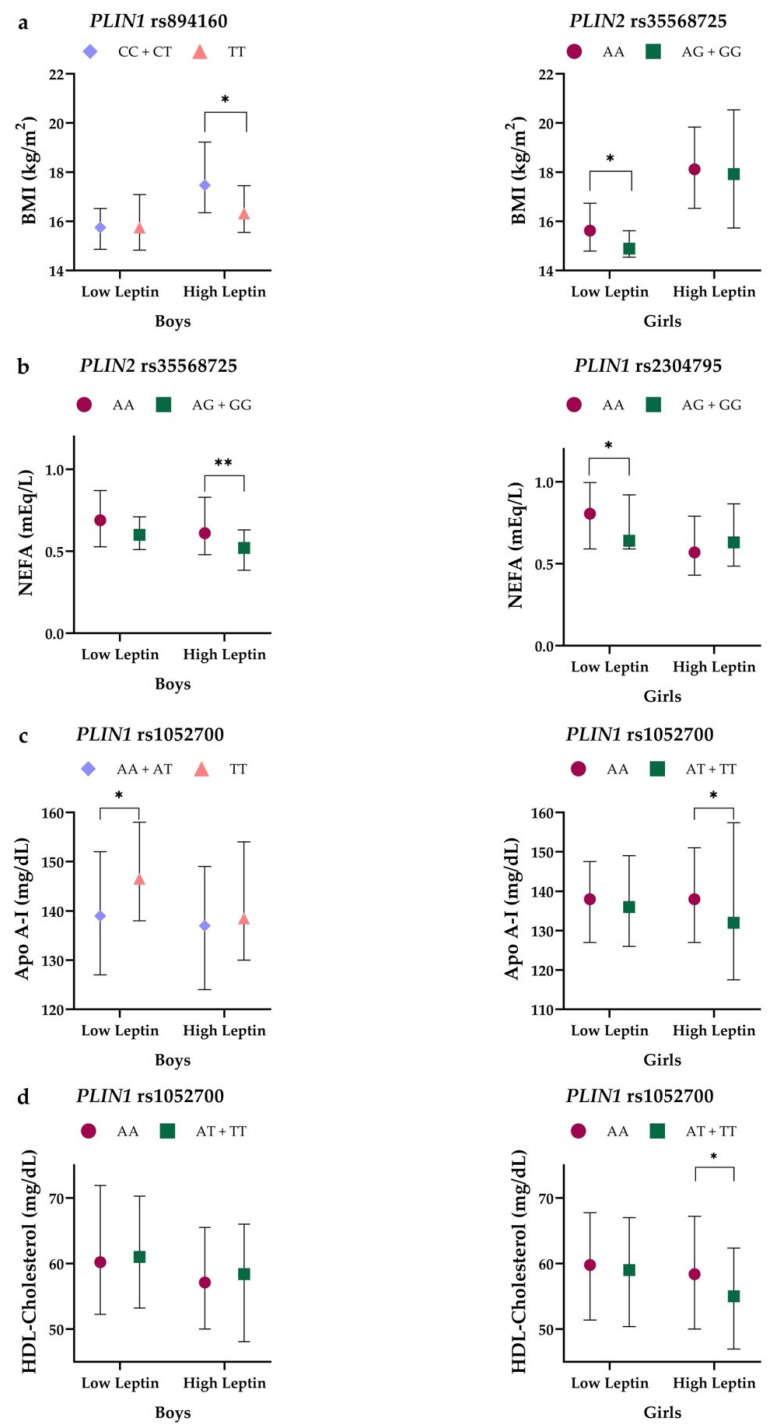
(**a**) BMI values of *PLIN1* rs894160 and *PLIN2* rs35568725 genotypes in boys and girls according to levels of leptin; (**b**) NEFA levels of *PLIN1* rs2304795 and *PLIN2* rs35568725 genotypes in boys and girls by leptin levels; (**c**) Apo A-I levels of *PLIN1* rs1052700 genotypes in boys and girls by levels of leptin. (**d**) HDL-cholesterol levels of *PLIN1* rs1052700 genotypes in boys and girls by levels of leptin. Values are expressed as median and interquartile range. *p*-value: Mann–Whitney U test: * *p*-value < 0.05; ** *p*-value < 0.01.

To analyze whether the effect of the polymorphisms studied on BMI and lipid levels was mediated by leptin levels, the relationship of the SNPs (rs894160, rs2304795, and rs1052700 in *PLIN1*, and rs35568725 in *PLIN2)* with these parameters was investigated in children classified into two groups according to their plasma leptin levels, i.e., boys and girls with plasma leptin levels above or below their respective median value of leptin. As shown in Figure 1, we observed that the associations of *PLIN1* rs894160 and *PLIN2* rs35568725 with BMI (Figure 1a) and of *PLIN1* rs2304795 and *PLIN2* rs35568725 with NEFA (Figure 1b) were different in boys and girls with lower leptin levels compared to subjects with higher leptin concentrations. Significant associations were observed in boys with leptin levels above the median values, as well as in girls with leptin levels below the median leptin value. Additionally, a different association in boys and girls depending on leptin levels was observed concerning the influence of *PLIN1* rs1052700 on Apo-I levels (Figure 1c). The association of the *PLIN1* rs1052700 SNP with HDL-cholesterol levels (Figure 1d) was evident in girls with high leptin concentrations. Furthermore, the *PLIN1* rs894160 and *PLIN*2 rs35568725 SNPs were also associated with HDL-cholesterol levels depending on leptin concentration and sex (Supplementary Figure S1). No significant results were found when analyzing the association of the SNPs studied with triglyceride concentrations depending on leptin concentration.

## 4. Discussion

Aiming to further clarify the reasons behind reported differences in the association of perilipin polymorphisms with obesity and obesity-related parameters, we analyzed the most commonly studied SNPs in *PLIN1* (11482G > A (rs894160), 14995A > T (rs1052700), and 13041A > G (rs2304795)) and the SNP in *PLIN2* Ser251Pro (rs35568725), causing a missense mutation in exon 6, in a cohort of prepubertal children showing significant differences in plasma leptin concentration between boys and girls [30]. In this population, potential confounding factors are fewer than in the pubertal and adult population. In our analysis, sex-dependent differences were observed in the association of the SNP rs1052700 of *PLIN1* with Apo A-I concentrations. Furthermore, an association of the SNP rs35568725 of *PLIN2* was found with NEFA, HDL-cholesterol, and Apo A-I concentrations in boys, which was not observed in girls. No significant associations of the polymorphisms in *PLIN1* and *PLIN2* were observed with body weight, BMI, LDL-cholesterol, or Apo B. 

The association of *PLIN* SNPs with anthropometric traits and obesity has been described in studies in adults, including populations of different ethnic groups [10,11,31,32,33]. Although studies analyzing the association of *PLIN* SNPs with plasma lipid concentrations are scarce, some studies have also reported an association of these SNPs with triglycerides and HDL-cholesterol levels [10,15]. However, other studies have failed to detect association between these SNPs and obesity or obesity-related parameters [34,35,36,37,38,39]. Interaction of *PLIN* SNPs with nutritional factors may represent a plausible explanation for discrepancies among studies [16]. Another important issue is the sex-dependent association between SNPs at the *PLIN* locus and the obesity risk reported in adult populations [31,40] which may contribute to explaining divergent findings.

Few studies have analyzed the relationship of *PLIN* SNPs with obesity or obesity-related alterations in children [19,20]. The design of these studies differs from ours, as Deram et al. [19] analyzed the effect of *PLIN* gene variation on weight loss in children with obesity aged 7–14 year, while the study of Tokgöz analyzed their association with obesity in a case-control study including 206 children with obesity and 102 healthy controls [20], which complicates efforts to compare our findings, as we analyzed a general child population and the analysis is not performed in overweight/obese children.

Here, we described a different sex-based association, particularly concerning the SNP rs1052700 of *PLIN1* and the SNP rs35568725 of *PLIN2* in a cohort of children in which a different nutritional status by sex, as reflected by plasma leptin levels, had been previously described [30].

Differences in diet have been associated with variations in the effect of the PLIN polymorphisms on obesity and obesity-related parameters [21,22,23,24]. Diet-induced changes in body fat and energy metabolism may be responsible for a different nutritional metabolism status, and these changes affect leptin levels. We hypothesized that the differences found in the effect of the polymorphism between boys and girls would be associated with the fact that the boys and girls in our population had significantly different leptin levels, and that plasma leptin levels could modulate the association of the polymorphisms with NEFA concentrations, which conditions its association with BMI and lipid metabolism. The *PLIN1* rs894160 and *PLIN1* rs1052700 have been associated with changes in abdominal fat and blood NEFA levels that occur in weight loss [41], which may also suggest an influence on these associations exerted by changes in leptin levels associated with changes in body weight. 

In our study, when analyzing the effect of the SNPs studied in children grouped according to plasma leptin levels, we observed that the relationship of the polymorphisms with BMI, Apo A-I, and NEFA varied depending on leptin concentrations. The effect of leptin on adipocyte metabolism has been demonstrated, and both direct and indirect effects of leptin on adipocyte metabolism have been suggested [27]. As adipocytes express leptin receptors, leptin may influence adipocyte metabolism directly and, as adipocytes are insulin-responsive, leptin can also modify adipocyte metabolism indirectly [27]. 

As described previously, perilipin is a protein that coats lipid droplets (LDs) in adipocytes [42] and plays an important role in lipolysis as, upon activation by protein kinase A, phosphorylated perilipin translocates from the lipid droplet and allows HSL to hydrolyze the TG and release NEFA. An important triggering role of leptin has been suggested for this intracellular lipolytic pathway. Indeed, PLIN1, the most abundant protein associated with LDs [43], is highly expressed in white adipocytes [44], and lower PLIN1 expression is related to higher rates of lipolysis [43]. Additionally, *PLIN* genetic variants may affect the protein content and lipolytic rates of adipocytes. Mottagui-Tabar et al. linked the rs894160 polymorphism of *PLIN1* to perilipin content in the adipocyte and basal and noradrenaline-induced lipolysis, with the 11482A allele being associated with a decreased perilipin content and with an increase in lipolysis [4]. The rs35568725 polymorphism of *PLIN2* has also been associated with an alteration of the gene that affects lipolysis and which is related to lower concentrations of TG [17]. Thus, based on the hypothesis that leptin triggers the lipolytic pathway that leads to phosphorylated perilipin and stimulates lipolysis, we assume a different effect of the genetic variants of *PLIN*, which determine the perilipin content in the adipocyte and PLIN functionality, and on BMI and lipid metabolism depending on leptin levels. 

We should mention the lack of information regarding body composition as the main limitation of our study, as information on body fat might help us to understand differences on plasma leptin levels between boys and girls. An inherent limitation of all cross-sectional studies is the inability to demonstrate causality. Therefore, further studies are needed to confirm the causal nature of these associations.

## 5. Conclusions

Based on our results, we may conclude that the association of the *PLIN1* and *PLIN2* polymorphisms with BMI, NEFA, and Apo A-I concentrations seems to be modulated by plasma leptin levels in prepubertal children. Our data may contribute to elucidate the discrepancies observed in previous studies analyzing these associations in adult populations. Our data allow us to speculate that different plasma leptin concentration by sex through life, affecting the relationship of the *PLIN* SNPs with cardiovascular risk factors, such as BMI and lipid profile, may contribute to explaining the different predisposition to cardiovascular disease depending on sex across life. Further studies analyzing these aspects in other groups of age should be performed.

## Figures and Tables

**Table 1:** Characteristics (means ± SD) of the population studied.

	Overall (n = 1264)	Boys (n = 633)	Girls (n = 631)	*p*-Value ^1^
Age (years)	7.2 ± 0.6	7.2 ± 0.6	7.2 ± 0.6	0.531
BMI (kg/m^2^)	17.0 ± 2.4	16.9 ± 2.4	17.0 ± 2.5	0.637
TC (mg/dL)	183.0 ± 28.6	182.6 ± 28.5	183.4 ±28.7	0.238
TG (mg/dL)	72.9 ± 26.3	71.4 ± 25.4	74.4 ± 27.2	0.014
LDL-C (mg/dL)	109.1 ± 27.3	108.1 ±27.7	110.0± 27.0	0.029
Apo B (mg/dL)	70.3 ± 15.0	69.2 ± 15.0	71.3 ± 15.0	0.001
HDL-C (mg/dL)	59.4 ± 13.3	60.2 ± 13.2	58.7 ± 13.4	0.021
Apo A-I (mg/dL)	137.0 ± 19.1	138.4 ± 19.1	135.5 ± 19.1	0.007
NEFA (mEq/L)	0.70 ± 0.28	0.68 ± 0.27	0.72 ± 0.30	0.074
Leptin (ng/mL)	6.58 ± 8.00	4.65 ± 6.43	8.60 ± 8.97	0.000

^1^*p*-value: Mann–Whitney U test. Abbreviations are as follows: BMI, body mass index; TC, total cholesterol; TG, triglycerides; LDL-C, low-density lipoprotein cholesterol; Apo B, apolipoprotein B; HDL-C, high-density lipoprotein cholesterol; Apo A-I, apolipoprotein A-I; NEFA, non-esterified fatty acids.

**Table 2:** Genotypic and allelic distribution of the SNPs studied in *PLIN1* and *PLIN2*.

Gene	SNP	Genotype	% (n)	Allele	%
*PLIN1*	rs894160	CC	49.4 (625)	C	70.5
		CT	42.1 (532)	T	29.5
		TT	8.5 (107)		
	rs1052700	AA	44.7 (557)	A	66.7
		AT	43.6 (544)	T	33.5
		TT	11.7 (146)		
	rs2304795	AA	34.1 (455)	A	61.0
		AG	47.4 (632)	G	39.0
		GG	13.2 (176)		
*PLIN*2	rs35568725	AA	88.1 (1110)	A	93.8
		AG	11.5 (145)	G	6.2
		GG	0.4 (5)		

**Table 3:** BMI and lipid profile values (means ± SD) by genotype for *PLIN1* and *PLIN2* SNPs in boys and girls.

**Boys**			**BMI (kg/m^2^)**	**TG (mg/dL)**	**HDL-C (mg/dL)**	**APO A-I (mg/dL)**	**NEFA (mEq/L)**
*PLIN1*	rs894160	CC (n = 306)	17.0 ± 2.4	69.1 ± 28.5	60.6 ± 12.7	137.9 ± 19.3	0.67 ± 0.25
		CT (n = 278)	16.9 −2.5	74.2 ± 28.8	59.8 ± 13.7	138.1 ± 18.7	0.69 ± 0.29
		TT (n = 49)	16.6 ± 1.9	66.5 ± 22.3	60.2 ± 13.1	143.2 ± 17.6	0.70 ± 0.27
		*p-value*	ns	ns	ns	ns	ns
	rs1052700	AA (n = 270)	17.0 ± 2.4	70.2 ± 23.5	60.2 ± 13.5	137.7 ± 18.9	0.66 ± 0.28
		AT (n = 282)	16.9 ± 2.3	72.4 ± 28.3	59.8 ± 12.8	137.6 ± 19.4	0.69 ± 0.27
		TT (n = 71)	16.9 ± 2.3	70.9 ± 21.0	62.1 ± 13.7	143.6 ± 17.6	0.69 ± 0.21
		*p-value*	ns	ns	ns	0.013 ^a^	ns
	rs2304795	AA (n = 228)	16.8 ± 2.4	69.6 ± 23.4	59.6 ± 14.0	136.8 ± 19.2	0.68 ± 0.28
		AG (n = 317)	17.0 ± 2.4	71.0 ± 27.4	60.9 ± 12.4	139.7 ± 18.9	0.67 ± 0.25
		GG (n = 88)	17.3 ± 2.3	75.7 ± 21.7	59.5 ± 13.1	137.8 ± 18.2	0.71 ± 0.32
		*p-value*	ns	0.008 ^b^	ns	ns	ns
*PLIN2*	rs35568725	AA (n = 549)	17.0 ± 2.4	71.0 ± 25.2	60.9 ± 13.2	139.3 ± 18.9	0.69 ± 0.28
		AG + GG (n = 80)	16.9 ± 2.7	72.1 ± 26.5	56.1 ± 12.0	133.2 ± 18.6	0.57± 0.19
		*p-value*	ns	0.767	0.005 ^c^	0.009 ^c^	**0.002 ^c^**
**Girls**							
*PLIN1*	rs894160	CC (n = 312)	16.8 ± 2.6	73.2 ± 29.6	58.7 ± 13.1	135.8 ± 19.1	0.73 ± 0.29
		CT (n = 250)	17.1 ± 2.5	75.0 ± 24.1	59.3 ± 13.8	135.8 ± 18.5	0.69 ± 0.31
		TT (n = 57)	17.3 ± 2.3	73.8 ± 19.8	58.5 ± 10.8	136.4 ± 20.1	0.79 ± 0.30
		*p-value*	ns	ns	ns	ns	ns
	rs1052700	AA (n = 281)	16.8 ± 2.5	75.9 ± 31.6	59.5 ± 14.0	137.9 ± 19.3	0.74 ± 0.31
		AT (n = 257)	17.1 ± 2.6	72.5 ± 21.3	58.6 ± 12.6	135.3 ± 18.3	0.70 ± 0.30
		TT (n = 75)	17.4 ± 2.1	73.1 ± 24.0	57.2 ± 12.5	130.2 ± 19.0	0.69 ± 0.26
		*p-value*	ns	ns	ns	0.006 ^a^	ns
	rs2304795	AA (n = 221)	17.0 ± 2.6	74.8 ± 25.4	58.9 ± 13.8	137.7 ± 17.3	0.73 ± 0.27
		AG (n = 310)	16.9 ± 2.5	74.1 ± 28.7	58.9 ± 13.0	135.0 ± 20.0	0.72 ± 0.30
		GG (n = 87)	17.0 ± 2.4	71.6 ± 22.3	58.9 ± 12.5	134.1 ± 18.9	0.69 ± 0.34
		*p-value*	0.881	0.551	0.813	0.304	0.175
*PLIN2*	rs35568725	AA (n = 550)	17.0 ± 2.5	74.4 ± 27.1	58.9 ± 13.0	135.8 ± 18.9	0.72 ± 0.30
		AG + GG (n = 68)	16.6 ± 2.7	70.2 ± 21.9	58.7 ± 14.5	136.4 ± 19.3	0.72 ± 0.30
		*p-value*	ns	ns	ns	ns	ns

*p*-value: Mann–Whitney U test; ^a^ AA + AT vs. TT; ^b^ AA + AG vs. GG; ^c^ AA vs. AG + GG. Abbreviations are as follows: ns, not significant; BMI, body mass index; TG, triglycerides; HDL-C, high-density lipoprotein cholesterol; Apo A-I, apolipoprotein A-I; NEFA, non-esterified fatty acids.

## Data Availability

Not applicable.

## Supplementary Materials

The following supporting information can be downloaded at: https://www.mdpi.com/article/10.3390/nu14153072/s1, Figure S1: (**a**) HDL-cholesterol levels of *PLIN1*rs894160 genotypes in boys and girls according to levels of leptin; (**b**) HDL-cholesterol levels of *PLIN2*rs35568725 genotypes in boys and girls by leptin levels. Values are expressed as median and interquartile range. *p*-value: Mann–Whitney U test: * *p*-value< 0.05; ** *p*-value < 0.01.
