# Supplementary material for: Sex-Dependent Mediation of Leptin in the Association of Perilipin Polymorphisms with BMI and Plasma Lipid Levels in Children
Source: Nutrients. 2022 Jul 26;14(15):3072. doi: 10.3390/nu14153072 (PMC9332311; doi:10.3390/nu14153072)
Supplement: Supplementary file 1 [file nutrients-14-03072-s001.zip › nutrients-1776257-supplementary.pdf]

nutrients

Supplementary Materials

Sex-Dependent Mediation of Leptin in the Association of Perilipin Polymorphisms with BMI and Plasma Lipid Levels in Children

Claudia Vales-Villamarín [1], Jairo Lumpuy-Castillo [2], Teresa Gavela-Pérez [3], Olaya de Dios [1], Iris Pérez-Nadador [1], Leandro Soriano-Guillén [3] and Carmen Garcés [1,*]

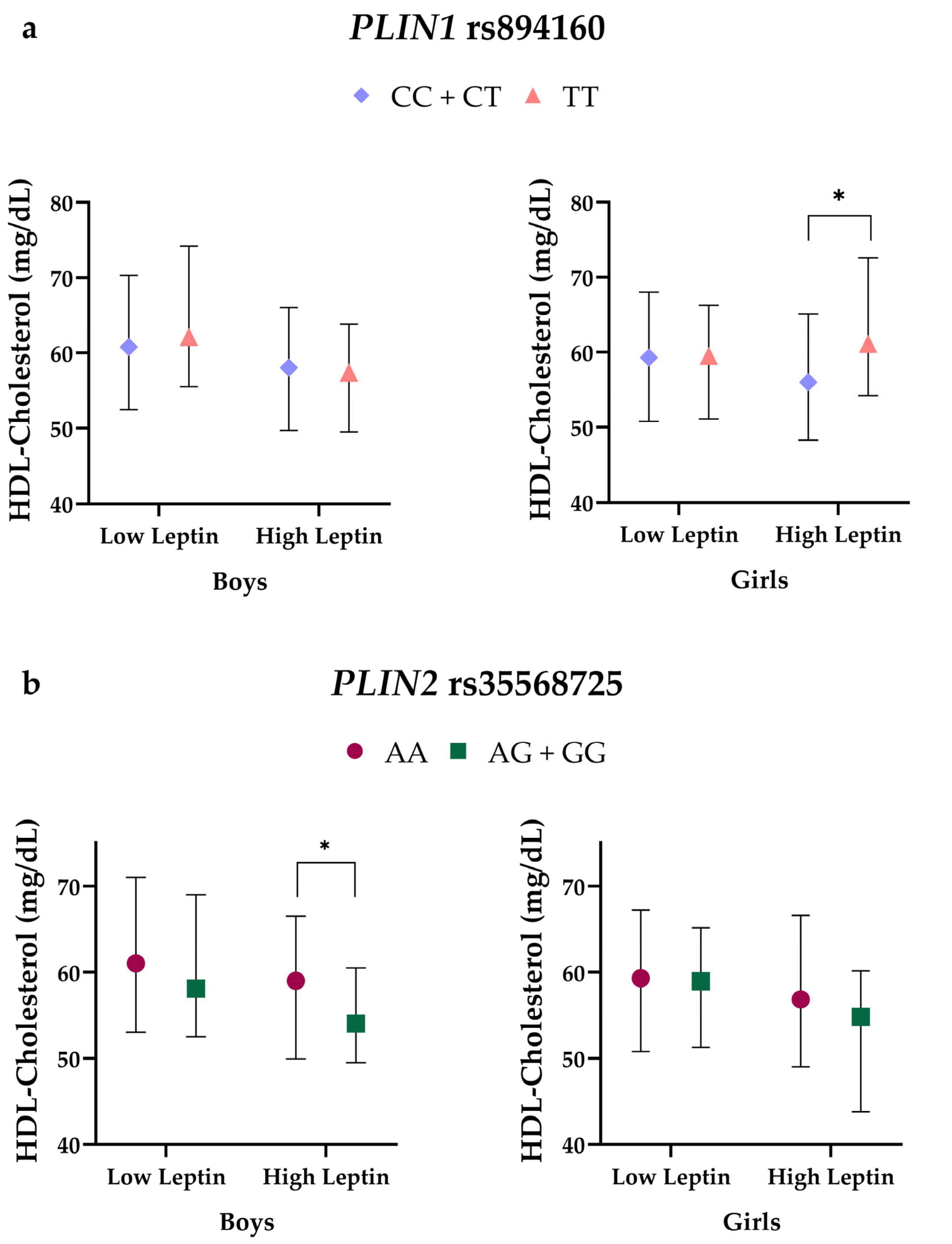

Figure S1. (**a**) HDL-cholesterol levels of *PLIN1*rs894160 genotypes in boys and girls according to levels of leptin; (**b**) HDL-cholesterol levels of *PLIN2*rs35568725 genotypes in boys and girls by leptin levels. Values are expressed as median and interquartile range. *p*-value: Mann–Whitney U test: * *p*-value< 0.05; ** *p*-value < 0.01.
